# Informal Caregiving and Health in Middle and Late Adulthood

**DOI:** 10.1093/geroni/igaa057.1955

**Published:** 2020-12-16

**Authors:** Joy Bohyun Jang, Sandra Tang

**Affiliations:** University of Michigan, Ann Arbor, Michigan, United States

## Abstract

With average life expectancy increasing, informal caregiving has become increasingly common among aging families. Much of the research in this area suggests that informal caregiving is associated with negative psychological and physical health outcomes for the caregiver. However, there is an emerging body of work indicating that these negative associations may be overstated, and that the associations may vary by gender and race. Using NLSY79 who completed Age 50 Health Module (n=7,844), we will examine how informal caregiving is associated with health in mid/late adulthood and how the association varies by race and gender of the caregiver. Preliminary results showed that caregivers (10.6% of the sample) were less likely to report good health than non-caregivers (OR=0.43, p<0.001) but African-American caregivers were more likely to report good health than other racial groups (OR=1.43, p<0.05). Our findings will contribute to better understanding of the role of informal caregiving in older adults’ health.

